# Feline Adenovirus Isolate Shows Silent Nucleotide Alterations, Alternative Receptor/Coreceptor Binding, High Resistance to Disinfectants and Antiviral Drugs, as Well as Immunomodulation

**DOI:** 10.3390/ani14233502

**Published:** 2024-12-04

**Authors:** Katalin Réka Tarcsai, Krisztián Bányai, Krisztina Bali, Anna Anoir Abbas, Valéria Kövesdi, József Ongrádi

**Affiliations:** 1 Doctoral School, Semmelweis University, 1085 Budapest, Hungary; kata.tarcsai@gmail.com (K.R.T.); abbas.anna@semmelweis.hu (A.A.A.); 2 Pathogen Discovery Group, HUN-REN Veterinary Medical Research Institute, 1143 Budapest, Hungary; krisz.bali@gmail.com; 3 Department of Pharmacology and Toxicology, University of Veterinary Medicine, 1078 Budapest, Hungary; 4 Szentágothai Research Centre, University of Pécs, 7622 Pécs, Hungary; 5 Department of Laboratory Medicine, Medical School, University of Pécs, 7622 Pécs, Hungary; 6 Department of Microbiology and Infectious Diseases, University of Veterinary Medicine, 1078 Budapest, Hungary; 7 Department of Preventive Medicine and Public Health, Semmelweis University, 1085 Budapest, Hungary; kovferne@gmail.com; 8 Department of Transfusion Medicine, Semmelweis University, 1085 Budapest, Hungary

**Keywords:** feline adenovirus, whole-genome sequencing, receptors, physico-chemical effects, antiviral therapy, cytokine production

## Abstract

Adenovirus (AdV) infection has been rarely documented in cats and other felids. Partial sequences of the hexon and fiber genes of a Hungarian feline adenovirus isolate (FeAdV isolate) showed a close relationship to human AdV (HAdV) type C1. Further molecular and biological characterization is reported here. Whole-genome sequencing revealed two silent mutations in the genome of the FeAdV isolate compared to a HAdV-C1 reference strain (at positions 14,096 and 15,082). Competitive antibody binding to the Coxsackie–adenovirus receptor and α_v_β_3_ and α_v_β_5_ integrin coreceptors inhibited the binding of the FeAdV isolate in different cell lines, but residual infections suggested alternative entry routes. The FeAdV isolate was found to be more sensitive to heat, low pH and detergents, but more resistant to alkaline and free chlorine treatments, as well as to ribavirin, stavudine and cidofovir treatments, than other human AdV types. We observed a suppression of IL-10 and TGF-β1 production during the entire course of viral replication. This immunomodulation may restore intratumoral immunity; thus, the FeAdV isolate could serve as an alternative oncolytic vector. Collectively, our results support that the Hungarian FeAdV isolate is a variant of common HAdV-C1. The cohabitation of cats with humans might result in reverse zoonotic infection. Felids appear to be susceptible to persistent and productive adenovirus infection, but further studies are needed to better understand the clinical and epidemiological implications.

## Simple Summary

Traditionally, adenoviruses are considered highly host-specific pathogens, although evidence of spillover events is continuously accumulating. In this study, we characterized a feline adenovirus isolate, which was hypothesized to have originated from a human adenovirus. We analyzed the viral genome sequence, the viral receptor usage and the susceptibility to various physico-chemical effects and antiviral drugs. The obtained information may help implement more stringent preventive and control measures in any environments where humans and cats are mutually exposed to adenovirus infections of the other host species.

## 1. Introduction

Adenoviruses (AdVs) are medium-sized, non-enveloped, double-stranded, linear DNA viruses that infect hosts in a wide range of vertebrates from Amphibia to humans [1]. Human adenoviruses (HAdVs) are grouped in the most intensively studied genus; in this paper, we refer to their characteristics unless otherwise stated. Their icosahedral capsid encloses a nucleoprotein core (~34,000 to 37,000 bp nucleotides). Numerous copies of virus-encoded major capsid proteins (hexon, penton and fiber) are expressed on the surface of the virion [2]. Several non-structural AdV polypeptides identified in infected cells regulate virus replication and adapt cellular machinery to support virus replication. In the genome of HAdV-1, altogether, 53 coding regions and several non-coding motifs have been determined [3]. The size and structure of both polypeptide-coding regions and non-coding motifs might vary in different species, showing different rates of homology and conserved motifs. These are used to determine phylogenetic relationships between AdV types, and conserved regions are used for PCR to detect AdV in specimens for diagnostic purposes and in search of novel species in new hosts. The virions are very compact and extremely stable in the environment [4]. The information on the effect of physico-chemical agents has been established for individual species without systematic comparison. Determining the physical stability of virions allows for safe handling and preservation of the infectivity of viral preparations if needed [5]. Most primary infections do not require chemotherapeutic intervention. AdV reactivation resulting in a high titer of infectious particles and consequent severe clinical conditions in the presence of an immunosuppressed state has to be treated with specific drugs. In vitro and in vivo trials have shown that HAdV types are sensitive to various antiviral agents depending on species and type. Cultivation of a novel AdV is a prerequisite to establish the range of effective antiviral drugs [6].

The AdV infection process is well known. In the majority of HAdV species (A, C, D and F), fiber knobs bind to Coxsackievirus–adenovirus receptors (CARs). The fiber protein contains three structural domains: the tail, the shaft and the knob. The tail is the binding site for the penton-base vertices. The shaft shows various lengths between HAdV types, resulting in different flexibilities of the fiber and differences in the interactions with host cells [2]. HAdV-F40, B species and some D species types bind to the CD46 receptor, desmoglein-2 (DSG2) and sialic acid, and G types bind to CAR and polysialic acid [7,8]. Internalization is initiated by the binding of penton bases to α_v_β_3_ or α_v_β_5_ integrin molecules. In their interactions, the arginine–glycine–aspartic acid (RGD) motif of the penton base and the LDV motif (leucine–aspartic acid–valine) play a role in triggering virus uptake by chlathrin-mediated receptor endocytosis. Several alternative attachment receptors have been identified [2,9], but less attention has been paid to their pathophysiological significance. Hexon proteins elicit species-specific immunity, while fiber proteins are the major determinants of tropisms and confer type-specific immunity [2].

The rapidly growing number of species in the *Adenoviridae* family are divided into five genera and a pending new genus. Among them, mastadenoviruses infect a wide range of mammals, including wild and domestic animals, as well as humans [10]. An AdV species usually has a narrow host range, restricted to one host or some closely related species, but shared adenovirus species in distantly related hosts have also been reported. Evidence implies that AdVs have been co-evolving with their hosts [10]. HAdV species show high homology at the nucleotide level. In the last several decades, multiple assumed host switches of AdVs have been described, e.g., between monkeys, apes and humans and other species [2,10,11]. Due to genetic variability, mutants can display different tissue or host tropisms, as well as increased virulence.

Human adenoviruses (HAdVs) are grouped into species A to G, comprising 51 serotypes and newly discovered genotypes. Altogether, more than 110 types have been described. In vivo molecular detection of putative genotypes was not followed by isolation in the majority of cases. A whole-genome sequence released in a public sequence database is the prerequisite for identifying a new AdV type and its inclusion of the new type in the International Committee on Taxonomy of Viruses (ICTV) [12].

HAdVs can acutely infect children, usually causing self-limiting diseases. Independently of each other, HAdVs, especially species C types (HAdV-C1, -C2, -C5 and -C6 serotypes) can establish life-long latency in lymphocytes, from which they can reactivate and consequently spread to numerous cell types and organs, causing systemic infection. These occur mainly in immunocompromised conditions, e.g., during cell or organ transplantation and AIDS. These opportunistic infections can be fatal, in spite of the species-specific immunity of the host. The molecular mechanism of HAdV reactivation and immune modulation has not been elucidated completely, but the balance of pro- and anti-inflammatory cytokines and chemokines plays a key role in the pathogenesis. Along with mobilization of innate immunity, rapid secretion of cytokine proteins is triggered in consecutive phases of the viral life cycle [2]. The cytokine/chemokine pattern might be species-dependent. The large number of genetically modified virus particles introduced in the body as therapeutic or vaccine AdV vectors elicit cytokine storm syndrome [1,13]. The regulation of the concerted action of mediator release is known only partially. Besides opportunistic infections, HAdVs can directly transactivate HIV replication in vitro [14] and in vivo, resulting in rapid progression of AIDS [15].

Recombinant human and animal adenoviruses carrying genes of heterologous microbes have been used in prophylactic immunization against infectious diseases, e.g., SARS coronavirus 2, as a recent case [16]. For decades, there have been several trials to use recombinant human and animal AdVs as anticancer therapeutic tools, especially in patients with prostate cancer (PCa). This cancer is the second most common cancer in men, its incidence is increasing and tumor-related death rate is expected to more than double by 2040. Recurrences and late-stage tumors are resistant to conventional chemo- and radiotherapy [17,18]. Several PCa cell lines displaying a wide range of genetic and phenotypic differences are used to study the effect of confounding factors, which might also influence the antitumor efficacy of AdV gene therapy [18,19]. New target cells are continuously required for biological, therapeutic and vector studies. Here, we provide a recently established PCa cell line.

At present, no ideal small-animal model (e.g., rodents) exists to study the molecular immunological pathomechanisms of different HAdV types, as well as their interactions with other microbes. In rodent models, AdV cannot replicate to a level observed in susceptible cell cultures. The feline AIDS model might open a new path in that direction. Until the discovery of the feline immunodeficiency virus (FIV) in 1987 [20], minimal attention was paid to the cat as a putative small-animal model [21]. Adenovirus-like particles in wild felids were detected occasionally. A captive leopard (*Panthera pardus*) in India presented intranuclear inclusion bodies and hepatitis [22]. In a domestic cat (*Felis catus*) co-infected with feline leukemia virus (FLV), AdV infection in endothelial cells was detected by light and electron microscopy [23]. These observations were not followed by virus identification and experimental studies. The importance of the HIV–AdV interaction in humans led us to study AdV infection in cats. Due to the lack of adequate feline-origin reagents, the purified hexon antigen of HAdV-C1 was used in a home-made ELISA in the initial seroepidemiological study to screen free-roaming cats. Seropositivity rates were 9.8–20.3% in European cats and 26% in cats sampled in the USA [24]. A similar survey of randomly selected feline sera in a Central European country showed 25% seropositivity, and among the six retrovirus-infected cats, three males with outdoor access were AdV-seropositive [25]. Later, FeAdV isolate-infected cells were used for screening antibodies by indirect immunofluorescence microscopy. In a kennel located in Delaware, USA, 80% seropositivity was found [9]. Furthermore, serial passage of FIV in experimental cats also accidentally transmitted AdV infection [24]. PCR screening of feces and saliva from many healthy and FIV-infected cats revealed the long-term shedding of AdV in one FIV-infected animal [26]. Co-cultivation of fecal samples in HeLa cells resulted in the isolation of an infectious agent [27], which was verified as an adenovirus and denominated as the feline adenovirus (FeAdV) isolate. The isolate was submitted to the American Type Culture Collection (ATCC catalogue number: VR-1890). Sequencing its hexon and fiber genes showed its relationship to a Central European HAdV-C1 (isolate 1038, genotype D11) [9,28]. The original name in the ATCC was Feline adenovirus 1, but according to recent whole-genome sequencing data available in GenBank, it was renamed Human adenovirus 1 and Feline isolate of HAdV-1. The feline AdV isolate replicated in many human and mammalian cell lines, except in CAR-negative rodent cell cultures. A cell line of human origin with low integrin expression (MEWO) was semipermissive for the FeAdV isolate. Lymphocytes and monocytes, although they were infected, did not support virus replication [9]. The close relationship between HAdV-C1 and the FeAdV isolate might result in interspecies transmission in both directions. A case report from Japan showed that a one-year-old girl and her cat had the same AdV resembling FeAdV isolate. The hexon and fiber genes of FeAdV isolate and those detected in Japan shared 100% and 97% identity at the amino acid level, respectively [29]. Another report from Brazil showed that 1 of 468 human upper respiratory tract specimens contained AdV sequences. These showed 100% nucleotide homology to the hexon gene of the FeAdV isolate counterpart [30]. These reports suggest that FeAdV isolate occurs, even if rarely, worldwide, and that it is genetically related to some HAdVs.

DNA from paraffin-embedded tissues of a cat with suspected disseminated AdV infection [23] was extracted and subjected to AdV-specific PCRs (both hexon- and DNA-dependent DNA polymerase gene fragments). Phylogenetic analyses showed that the hexon sequences are related to squirrel AdV with 80% nucleotide identity. On the contrary, DNA polymerase fragment sequences showed the closest relationship (90%) to the sequence fragment of the Chinese horseshoe bat (*Rhinolophus sinicus*) [31]. A recent AdV surveillance in wild carnivores in Brazil detected AdV sequences in different tissues (including lymph nodes) of a road-killed ocelot (*Leopardus pardalis*) by amplifying DNA polymerase. The obtained sequence presented a low deduced amino acid similarity (71.8%) to the closest AdV, identified in a pinniped from USA. It could be a novel species, denominated as ocelot adenovirus-1 [32]. Systemic infection suggests that this virus replicated in the ocelot. This is the third molecularly identified AdV among felids. These cases show that cats and other felids can harbor AdVs of different origin.

Scarce data [9,22,23] show that adenovirus infection in cats induces transient or lethal hepatic failure. Simultaneous FIV [9,25,26] or FLV infection [23] might predispose to AdV carriage and reactivation or vice versa. The clinical consequences of FeAdV infection in cats and humans are not known; they ought to be investigated at individual and population levels, and their immunomodulatory effects should also be focused on. Data on therapy, prevention, proper handling by disinfection, and diagnostic preparations for human and veterinary laboratory practices are needed.

Taken together, missing information on FeAdVs prompted us to extend the characterization of the Hungarian isolate of feline-origin AdV. Whole-genome sequencing (WGS) is a prerequisite for considering this virus as a putative gene therapy vector. In this study, we carried out WGS, and the results strengthened the relationship between the FeAdV isolate and HAdV-C1. Also, a new prostate cancer cell line established in our laboratory showed high permissivity to FeAdV infection. We determined here the physico-chemical effects of antiviral drugs on the infectivity of the FeAdV isolate. Sensitivity of FeAdV isolate to drugs showed differences from that of other HAdV types. In this study, basic immunoregulatory effects were also determined by quantitation of IL-10 and TGF-β_1_ secretion through the whole replication cycle of the FeAdV isolate. The results suggest that the FeAdV isolate suppresses constitutive secretion of both mediators during the whole course of the replication cycle.

## 2. Materials and Methods

### 2.1. Cells, Virus and Titration

Three cell lines were used: human cervical carcinoma cells (HeLa; obtained from Dr. György Berencsi, National Institute of Public Health, Budapest, Hungary), human embryonal kidney cells (HEK-293; obtained from Dr. László Rosiwall, Department of Pathophysiology, Semmelweis University, Budapest, Hungary) and normal feline kidney epithelial cells (CRFK; obtained from Dr. Mauro Pistello, Retrovirus Center, University of Pisa, Pisa, Italy). They were maintained in Dulbecco’s Modified Eagle’s medium (DMEM) with 4.5 g/L glucose, L-glutamate and sodium pyruvate containing 10% heat-inactivated fetal bovine serum (FBS) and 1% penicillin–streptomycin (Lonza, Walkersville, MD, USA) (tissue-culture medium, TCM). All cultures in this complete medium were incubated in 5% CO_2_ at 37 °C. A prostate carcinoma cell line was established from the surgically removed tumor mass of a 56-year-old patient, and the new cell culture was named “ongjos” after the donor. An approximately 1 cm^3^ cancer fragment immersed in TCM containing 20% FBS was minced and subsequently treated with 0.05% trypsin plus 1 mM ethylenediaminetetraacetic acid (EDTA) solution. Cells were collected by low-speed centrifugation (1200 rpm, 4 °C, 10 min). Trypsinization and centrifugation were repeated three times. Next, cells were aliquoted into tissue-culture flasks and plates containing 10 mL TCM plus 20% FBS and incubated in 5% CO_2_ at 37 °C. Over the following days, several cells could be seen to attach to the bottom and finally form a regular monolayer. From the monolayer cultures, passages were performed by the usual trypsinization method, splitting cultures in 1:2 or 1:3 ratios. The TCM contained 10% FBS. No testosterone was added to the cultures. Cells in plates were fixed by using 96% ethylene alcohol and stained with Giemsa or methylene blue solutions for 30 min. Photographs were taken using an Olympus microscope. Electron micrograms were prepared as described previously [9]. Ethical review and approval were waived for this study by the Department of Public Health, Semmelweis University, due to the personal agreement of the donor of the ongjos cells and the department.

Our FeAdV isolate was used in this study. HeLa cells in complete medium in 75 cm^2^ tissue-culture flasks were infected at a high multiplicity of infection (moi) and were harvested by low-speed centrifugation when the cytopathic effect (CPE) was shown to be almost 100%. Both supernatants and cell pellets were stored at −20 °C. On day 5 post-infection, the FeAdV titer in the clarified supernatant reached 4.0 × 10^4^ infectious units/mL (IU/mL). Aliquots of supernatants were purified and concentrated with a purification filter (ViraBindTM Adenovirus Purification Kit; Cell Biolabs, Inc., San Diego, CA, USA). The final concentration of the virus stock was 3.43 × 10^9^ infectious units/mL (IU/mL) as titered on HeLa cells. Cells and virus stocks were free of mycoplasma contamination and tested negative for bacterial endotoxin (LPS) [9].

Similarly to the production of virus stocks in HeLa cells, CRFK cells in tissue-culture flasks were infected with FeAdV. Subsequently, virus-containing supernatants were harvested by centrifugation and stored at −20 °C. Serial dilutions of the virus stock (denoted here as the CRFK-FeAdV isolate) were titrated on both HeLa and CRFK cells [9]. Its titer on HeLa cells was 3.3 × 10^3^ infectious units/mL, and that on CRFK cells was 3.7 × 10^4^ infectious units/mL. On the contrary, the FeAdV isolate produced in HeLa cells and titrated on CRFK cells resulted in 2.6 × 10^3^ IU/mL.

Virus replication was also quantitated by the presence of intracellular hexon antigens. It was visualized by immunoassay (Quick titer Adenovirus Titer Immunoassay Kit; Cell Biolabs, Inc., San Diego, CA, USA). This kit provides a quick and complete system to functionally titer virus infectivity, as described previously. The different cell cultures used in this study were infected with FeAdV isolate preparations and these were regarded as positive controls, while uninfected and untreated cells were used as negative controls. Unless otherwise stated, the FeAdV isolate produced on HeLa cells was used throughout this study [33,34].

### 2.2. Experimental Procedures

#### 2.2.1. Genome Sequencing of the FeAdV Isolate

The DNA of the FeAdV isolate was extracted from the supernatant of HeLa cells using the innuPREP Virus DNA/RNA Kit (Analytik Jena, Jena, Germany) according to the manufacturer’s instructions, and the concentration of purified DNA was measured with Qubit 2.0 equipment using the Qubit dsDNA BR Assay Kit (Thermo Scientific, Waltham, MA, USA). Nucleotide sequences were determined in parallel using two distinct barcodes to minimize the chance of mutations remaining in the final consensus sequence due to the applied sequencing chemistry. In brief, Illumina-specific DNA libraries were prepared using the Illumina^®^ Nextera XT DNA Library Preparation Kit (Illumina, San Diego, CA, USA) and the Nextera XT Index Kit v2 Set A (Illumina, San Diego, CA, USA), following the protocol described previously [35]. Libraries were purified using the Gel/PCR DNA Fragments Extraction Kit (Geneaid Biotech Ltd., Taipei, Taiwan). The concentration of the purified libraries was measured using the Qubit dsDNA HS Assay Kit (Thermo Scientific); then, the libraries were pooled and denatured. The denatured library pool at a final concentration of 1.5 pM was loaded onto a NextSeq 500/550 Mid Output flow cell and sequenced using an Illumina^®^ NextSeq 500 sequencer (Illumina). Sequence reads generated by the Illumina NextSeq 500 platform were assembled and analyzed by Geneious Prime (Biomatters Ltd., Auckland, New Zealand).

#### 2.2.2. Analysis of Viral Replication in Prostate Cancer Cells

A total of 4 × 10^4^ ongjos cells were aliquoted in 100 μL complete medium in wells of 96-well tissue-culture plates. Upon reaching confluency during incubation in 5% CO_2_ at 37 °C overnight, the media were replaced by FeAdV isolate and CRFK-FeAdV isolate stocks at 2 × 10^3^ IU/mL inputs and their 10-fold dilutions in 100 μL for 2 h in triplicate. Viruses were removed and cells were incubated in fresh complete medium for 7 days, when infectious virus titers were quantitated based on CPE.

#### 2.2.3. Analysis of Viral Attachment and Entry Receptors

HAdV high-affinity CAR, α_v_β_3_ and α_v_β_5_ integrin coreceptors were blocked with specific polyclonal and monoclonal antibodies (Anti-CXADR polyclonal antibody, Anti-integrin α_v_β_3_ monoclonal antibody and Anti-integrin α_v_β_5_ monoclonal antibody—all of the antibodies were produced in rabbits; Merck KGaA, Darmstadt, Germany). In 24-well tissue-culture plates, 2.5 × 10^5^ HeLa, CRFK and HEK-293 cells in 1 mL complete medium were seeded and incubated in 5% CO_2_ at 37 °C for 1 h. The cells were then incubated with antibodies at a final concentration of 2 μg/mL anti-CAR and 3 μg/mL anti-integrin in an orbital shaker at room temperature for 1 h. Several combinations of antibody mixtures were used: anti-CAR, anti-CAR + anti-α_v_β_3_, anti-CAR + anti-α_v_β_5_, anti-α_v_β_3_ + anti-α_v_β_5_ and anti-CAR + anti-α_v_β_3_ + anti-α_v_β_5_. Subsequently, the cells were infected with 100 μL virus stocks at 0.1, 1 and 10 moi. After 2 h of incubation in 5% CO_2_ at 37 °C, the antibody- and virus-containing media were decanted, and 1 mL fresh TCM was added to the cells. The infected cells without antibody treatment served as positive controls, while other cells without infection but with antibody treatment were regarded as negative controls. Samples were prepared in triplicate. Aliquots of mock-infected and infected cells were also treated with an irrelevant antibody to human herpesvirus 7 (RK4; Advanced Biotechnologies Inc., Columbia, MD, USA) [9]. The virus titer was quantitated by the intracellular hexon antigen content using immunochemistry at 48 h post-infection. Results are shown as % of virus content compared to untreated virus controls.

#### 2.2.4. Studies on the Physico-Chemical Effects on Viral Infectivity

In general, the effect of selected physico-chemical effects on FeAdV isolate infectivity was studied using HeLa cells and the FeAdV isolate at moi 1.

Heat inactivation: Heat inactivation of the FeAdV isoalate was carried out using 2.5 × 10^5^ IU/mL virus stock in 1 mL complete medium aliquoted in Eppendorf tubes. Altogether, 31 sample tubes were immersed in a water bath preheated to 56 °C (Falcon Instuments, srl., Treviglio, BG, Italy). In the first round of experiments, 100 μL samples were removed at 5, 10, 15, 20, 25, 30 and 35 min, followed by a second round in which samples were taken every minute between 0 and 5 min and collected in a 4 °C refrigerator. Subsequently, 100 μL from each treated sample was titrated on HeLa cells in triplicate, according to previous studies [5,36,37].

UV irradiation: UV-C at a wavelength between 200 and 270 nm results in pyrimidine dimerization resulting in DNA damage. Stocks of the FeAdV isolate containing 2.5 × 10^5^ IU/mL virus were exposed to UV irradiation using a low-pressure UV lamp (https://lumenet.hu/narva-lt-T8-18w-uv-c-g13-590mm-germicid-fenycső, accessed on 11 May 2022) at a 253.7 nm wavelength, 50 mW/m^2^, for 10, 15, 20, 25 and 30 min. Incubations were followed by sample collections every minute until 10 min. Samples were stored at 4 °C during the collection time, then HeLa cells were infected with 100 μL/well of the treated virus.

pH: Solutions with pH values between 1 and 14 were freshly prepared using 1 M NaOH and 37% HCl. They were sterile-filtered before use through 0.22 μm Millex-GP Filters (Merck Millipore, Burlington, MA, USA). The pH was double-checked using a digital pH meter (OP-211-1; Radelkis, Budapest, Hungary) and pH indicator paper (Merck KGaA). Then, 1 mL solutions at different pHs were added to 100 μL of the viral inoculum (1.5 × 10^5^) at 22 °C and 37 °C for 5 min. Subsequently, these solutions were neutralized to pH 7.4 using HCl or NaOH to prevent cell damage. Finally, HeLa cells were infected with 400 μL FeAdV isolate treated at different pHs.

Disinfectants—chlorine and 100% ethyl-alcohol: Two-fold dilution series were prepared from the two most commonly used skin and surface disinfectants. A stock solution at 40 g/L of NaClO (CAS no.: 7681-52-9) and a 650 mg/L stock solution of 0.4% alkyl-dimethyl-benzyl-ammonium chloride (CAS no.: 68424-85-1) diluted in 65.5% ethanol (CAS no.: 64-17-5) (100%, Merck KGaA) were prepared. Two-fold and 10-fold serial dilutions were prepared; subsequently, purified virus stocks were added to the dilutions for 5 min. Then, HeLa cells were infected with 100 μL/well of the treated virus.

Antiviral drugs: In 24-well tissue-culture plates, 2.5 × 10^5^ cells/well were seeded and incubated in 5% CO_2_ at 37 °C overnight. Three antiviral drugs used to treat HAdV infections were tested. Ribavirin is a guanoside analogue, cidofovir is a cytosine nucleotide analogue and stavudine (2′,3′-didehydro-3′-deoxythymidine) is a thymidine analogue. Stock solutions were prepared from lyophilized antiviral agents (Sigma-Aldrich, St. Louis, MO, USA) in complete DMEM (ribavirin: 50 mg/mL; cidofovir and stavudine: 10 mg/mL) and stored at −20 °C until use. In a preliminary experiment, cells were treated with 10-fold dilutions of the drugs to establish the wider range of effective concentrations. In the next phase of experiments, confluent HeLa cells were treated with 100 μL serially 2-fold diluted drugs: between 50 and 0.625 mg/mL of ribavirin, or between 10 and 0.3125 mg/mL cidofovir or stavudine, and these cell cultures were incubated in 5% CO_2_ at 37 °C for 1.5 h. The drugs were removed and replaced with 1 mL complete DMEM containing 10^9^ IU/mL FeAdV isolate. Until day 2, no toxic effect was detected. In the following experiment, cells were infected as above. At 2 and 24 h post-infection, the media of infected cells were decanted, and cells were treated with drug dilutions in 1 mL TCM for 2 h. In general, drugs and viruses were removed before the following drug treatment and infection, respectively.

Positive controls in all forms of treatment protocols contained virus stocks only, while negative controls were uninfected, untreated cells. Virus titer was determined by intracellular hexon content using immunochemistry at 48 h post-infection or drug treatment. The 50% inhibitory concentrations of the applied drugs (IC_50_s) were calculated. All experiments were carried out in triplicate.

#### 2.2.5. Quantitation of Cytokines

In 96-well plates, 4 × 10^4^ HeLa cells in 100 μL complete medium were seeded and incubated in the above conditions for one day. Half of the cell cultures were infected with the FeAdV isolate at 1 moi by adding virus stocks in 100 μL complete medium for synchronized infection. Mock-infected cells were treated with 100 μL medium. At designated time intervals (0, 2, 4, 6, 8,10, 24, 48, 72, 96 and 168 h), 100 μL medium was removed from the infected and mock-infected wells, in triplicate, and stored at −80 °C. When all samples were collected, the supernatant media were quickly melted, kept in ice-water, and the quantities of interleukin (IL)-10 and tumor growth factor (TGF)-β_1_ were determined by ELISA according to the instructions of the manufacturer (DuoSet ELISA Human Il-10 and Human TGF-β_1_ kits; R&D Systems Inc., Minneapolis, MN, USA). Serial dilutions of the standards between 31.2 and 2000 pg/mL supplied in the kits served as controls. Briefly, 100 μL diluted capture antibody was introduced into the wells at 22 °C and left overnight. After removing the unbound antibodies and washing the wells with 400 μL PBS containing 0.05% Tween-20 buffer three times, antibodies were blocked with 300 μL reagent diluent per well. After a single wash, 100 μL supernatant samples were introduced into the wells in triplicate. Serial dilutions of the standards, supplied by the kits, served as controls. After 2 h incubation at 22 °C and a single wash, 100 μL detection antibody was introduced into the wells at 22 °C for 2 h. Following a wash step, 100 μL streptavidin–HRP was added to the wells at 22 °C for 2 min. After a wash, again, 100 μL substrate solution plus Color reagent A mixture plus 100 μL Color reagent B was aliquoted at 22 °C for 20 min. The reaction was stopped by 2 N sulfuric acid. For quantitation of TGF-β_1_, Block buffer was used for blocking instead of Reagent diluent. OD values were measured at 450 and 540 wavelengths. The background was calculated by the differences between the two values.

### 2.3. Statistical Analysis

Experiments were carried out in triplicate. The data were expressed as means and standard errors of the means. Statistically significant differences were evaluated by a two-sample *t*-test (*p* < 0.05 was deemed to be significant).

## 3. Results

### 3.1. Whole-Genome Sequencing

**Figure 1:**
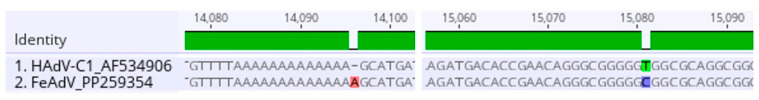
Sequence alignment showing SNPs at positions 14,096 and 15,081 in FeAdV isolate. For alignment purposes, FeAdV isolate nucleotide 15,082 was compared to HAdV-C1 nucleotide 15,081. Red: a nucleotide insertion, green to blue: a nucleotide change.

The consensus genome was determined by mapping against the compete genome sequence of the reference HAdV-C1 strain (accession no.: AF534906). The complete genome sequence of the FeAdV isolate (accession no.: PP259354) was 35,990 nucleotides (nt) long and showed 99.997% nucleotide identity with the HAdV-C1 reference strain. In the alignment, an adenine (A) was found to be inserted in the non-coding region at nt position 14,096 (Figure 1), and a thymine (T) was replaced by cytosine (C) with no consequent amino acid alteration in the penton-base RGD loop at nt position 15,082, which corresponds to nt 15,081 in the genome of HAdV-C1 (Table 1).

The fiber gene of the FeAdV isolate showed 100% homology with the corresponding HAdV-C1 gene (Table 2).

### 3.2. Replication of the FeAdV Isolate in Prostate Cancer Cells

**Figure 2:**
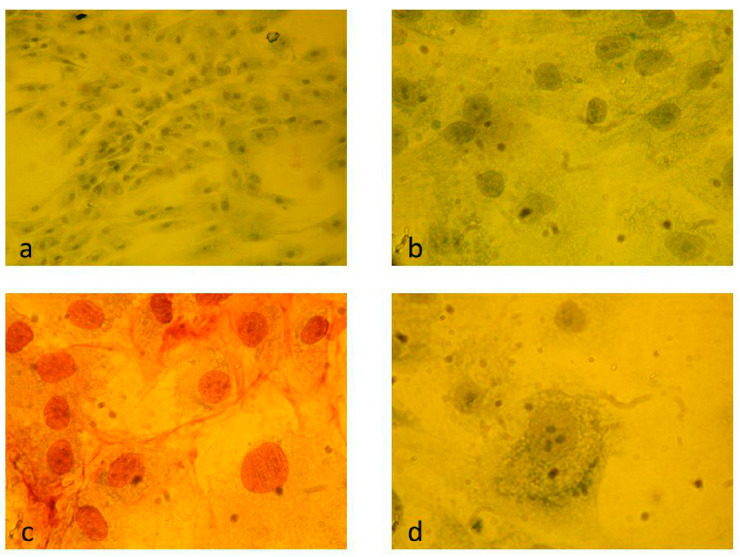
Uninfected ongjos cells. (**a**) Subconfluent monolayer with several elongated cells. Nuclei contain one or multiple nucleoli, day 3. (**b**) Higher magnification of the confluent monolayer. Nuclei contain dark nucleoli, day 7. (**c**) Torn monolayer. Apoptotic granules are in the cytoplasm of dying, non-colored cells, day 9. (**d**) Disintegrating cells with very large nucleoli, apoptotic granules in the cytoplasm, day 12. (**a**–**d**) Original magnification is 400× plus picture magnification. (**a**,**b**) Methylene blue staining).

The histology of the tumor mass showed that it was an adenocarcinoma, Gleason score 7, T3a, N0, Mx. The nuclei were very strongly androgen receptor-positive. Cell polymorphism was not characteristic; nucleoli could be seen in the nuclei of the cells. In culture, the cells formed a monolayer in tissue-culture flasks, as well as in 24-well and 96-well plates. Non-confluent cells were elongated as spindles, but at confluency they became rounded. One or more nucleoli could definitively be seen continuously during in vitro culture. Long cultivation (>12 days) without TCM change resulted in programmed cell death as detected by apoptotic granules in the cytoplasm (Figure 2).

**Figure 3:**
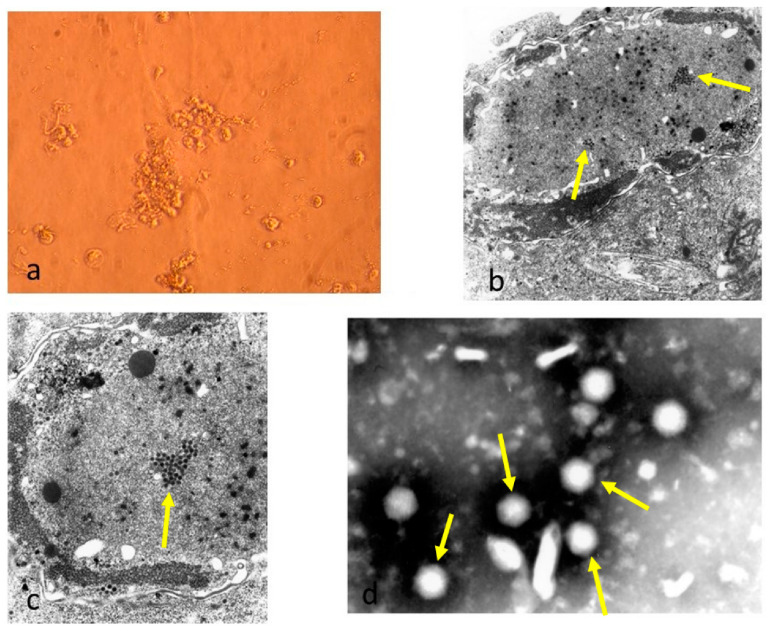
FeAdV isolate replication in ongjos cells. (**a**) Cytopathic effect of FeAdV isolate on day 5 (original magnification 400× plus picture magnification). (**b**) FeAdV isolate particles dispersed in the cytoplasm (original magnification 7000× plus picture magnification). (**c**) A pseudoarray of viral particles in the cytoplasm (original magnification 14,000× plus picture magnification). (**d**) Negatively stained viral particles from the supernatant of ongjos cells. Fibers could not be visualized (original magnification 50,000× plus picture magnification). Arrowheads point out virions (**b**,**d**) and a pseudoarray (**c**).

Both FeAdV isolate stocks induced gradually developing CPEs on the prostate cancer cells. CPE was the same as seen on several types of tissue-culture cells, e.g., HeLa, Vero, HEK-293, etc. Monolayer cells rounded up, clustered, and finally detached in the tissue-culture flask and disintegrated (Figure 3).

On day 7, the titer of the FeAdV isolate in the ongjos cells reached 4 × 10^4^ IU/mL, while the titer of the CRFK-FeAdV isolate was found to have reached 2 × 10^3^ IU/mL.

### 3.3. Determination of Cell Receptors of the FeAdV Isolate

**Figure 4:**
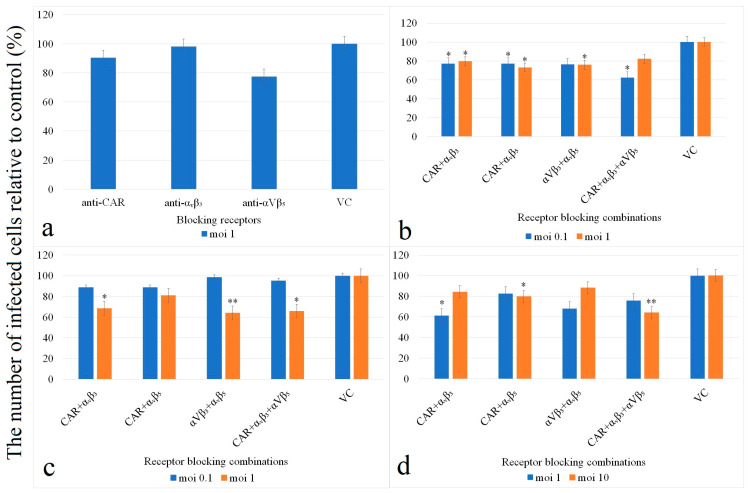
The effect of anti-Coxsackie–adenovirus receptor (CAR) and anti-α_v_β_3_ and anti-α_v_β_5_ antibodies on the relative number of virus-infected cells compared to the virus control (VC). (**a**) FeAdV isolate infection (moi 1) of Hela cells. (**b**) Combined antibody treatment of infected Hela cells (moi 0.1 and moi 1). (**c**) Combined antibody treatment of infected HEK cells (moi 0.1 and moi 1). (**d**) Combined antibody treatment of infected CRFK cells (moi 1 and moi 10). n = 3; * *p* < 0.05, ** *p* < 0.001.

Blocking either CAR or one of the integrin coreceptors on HeLa cells using the corresponding antibodies resulted in a considerable reduction in viral cell entry and consequently the ratio of hexon antigen-positive cells to antibody-free virus controls (Figure 4).

The strongest inhibition (near 23%) was detected when α_v_β_5_ coreceptors on HeLa cells were covered by antibodies at the time of infection. This competitive antibody binding to CAR or α_v_β_3_ decreased virus entry by 10% and 2%, respectively (Figure 4a). Several combinations of antibodies as well as different inocula (moi 0.1, 1 or 10) were applied on HeLa, HEK-293 and CRFK cells. Following infection of HeLa cells with FeAdV at moi 0.1 after receptor and coreceptor blocking, the ratio of antigen-positive cells decreased by almost 40% if CAR and both integrin molecules were blocked. The results were very similar after blocking CAR plus one of the coreceptors, or blocking coreceptors only; in each case, 23% fewer cells supported virus replication as compared to the virus control. In the same combinations but using a higher FeAdV isolate inoculum (moi 1), the reduction in the number of infected cells was between 18% and 27% (Figure 4b).

The highest inhibition (11.5%) of FeAdV isolate infection of HEK-293 cells at moi 0.1 was achieved by blocking CAR plus one of the integrin molecules at the same time (Figure 4c). In case of simultaneous blocking of the receptor and both coreceptors, or blocking of the coreceptors, we could not detect a significant suppressive effect. On the contrary, spectacularly less antigen-positive cells were determined upon infection with the FeAdV isolate at moi 1 applying the same treatment regimes as above. A greater than 34% decrease occurred when all three receptors and coreceptors, as well as only α_v_β_3_ and α_v_β_5_ molecules, were blocked before infection. Anti-CAR plus anti-α_v_β_3_ monoclonal antibodies diminished virus infectability by 32%, but treatment with both anti-CAR and anti-α_v_β_5_ antibodies hindered virus infection by less than 20%.

Infection of CRFK cells by a moderate FeAdV isolate inoculum (moi 1) after blocking CAR and α_v_β_3_ coreceptors resulted in the most significant drop (nearly 40%) in the number of infected cells (Figure 4d). Simultaneous blocking of both integrin coreceptors also resulted in a considerably smaller number (32%) of hexon antigen-positive cells. Competitive inhibition of CAR plus α_v_β_5_ decreased FeAdV isolate infection by 17% only. Simultaneous blocking of CAR and both coreceptors at the same time was not significant. Exposing cells to a very high inoculum (moi 10) after preceding competitive receptor and coreceptor blocking inhibited virus entry at the highest rate (36%). Simultaneous blocking of CAR and one of the integrin coreceptors as well as contemporary blocking of both coreceptors resulted in a similar lower ratio of hexon antigen-positive cells (12% to 20%) as compared to antibody-free virus controls.

### 3.4. Effect of Physico-Chemical Agents on the Infectivity of the FeAdV Isolate

#### 3.4.1. Heat Treatment

**Figure 5:**
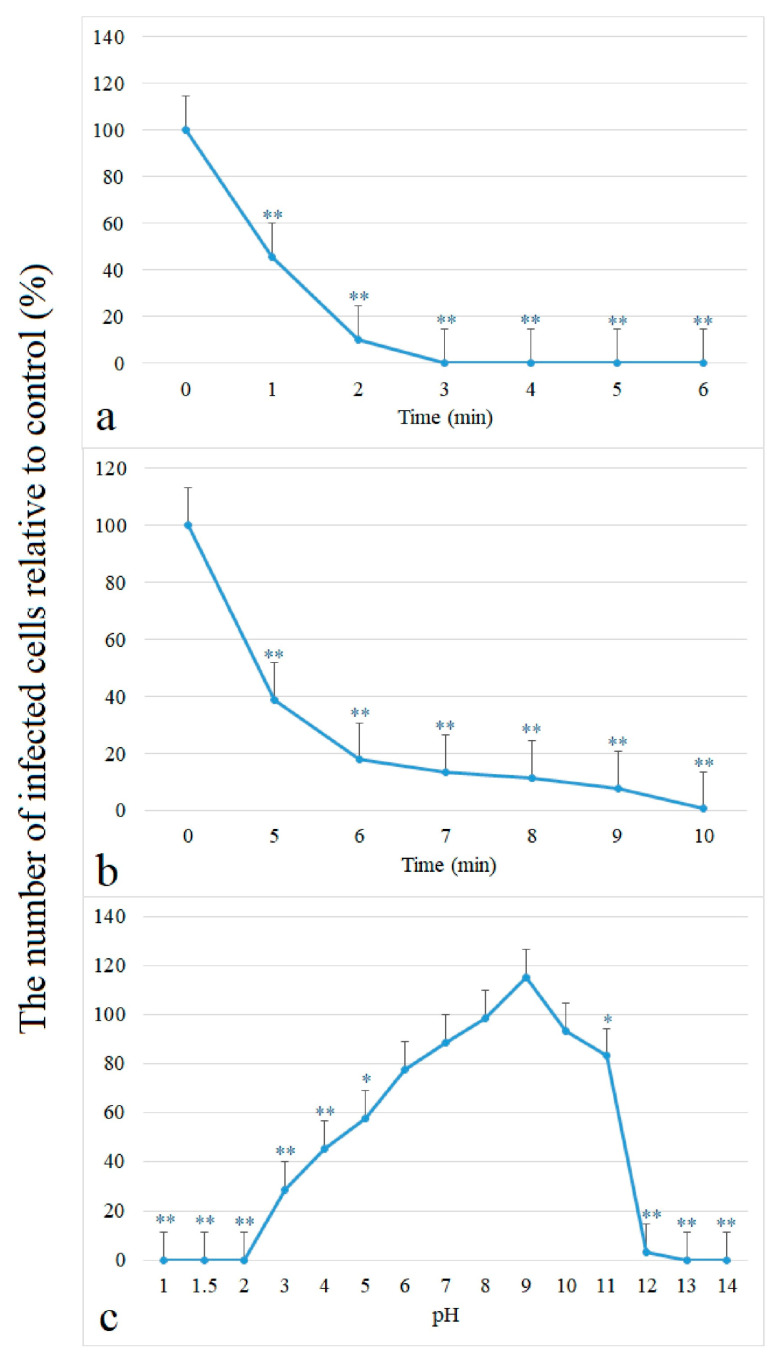
The effect of physico-chemical agents on residual virus infectivity assayed in HeLa cells. (**a**) Heat treatment at 56 °C. (**b**) UV treatment. (**c**) FeAdV isolate exposed to a pH range. n = 3; * *p* < 0.05, ** *p* < 0.001.

Adenoviruses are usually exposed to 56 °C for 5 and 30 min of heat treatment. Our preliminary results showed that the FeAdV isolate completely lost infectivity in HeLa cells after both time periods. In the following step, the FeAdV isolate was treated at 56 °C for gradually increasing times between 0 and 6 min. Infectivity decreased exponentially: after one min, it dropped by 54%, while 2 min of heat exposure reduced it by more than 90%. Three minutes of exposure resulted in a complete loss of infectivity: none of the HeLa cells contained hexon antigens when they were tested by immunochemistry (Figure 5a).

#### 3.4.2. UV Light

Studies on the UV sensitivity of the FeAdV isolate started with a wider time range: at 5 min and 30 min, because UV disinfection is generally applied for 30 min. Preliminary results indicated that 5 min UV exposure did not destroy all virus particles but 30 min of irradiation did. Between these two times, 10, 15, 20 and 25 min exposures were applied, and we found that exposure for 10 min or longer completely abolished infectivity. Presuming that the effective UV destruction could be between 5 and 10 min, aliquots of FeAdV isolate stock were irradiated gradually for between 5 and 10 min. The number of antigen-positive cells decreased by more than 60% after 5 min irradiation as compared to the non-irradiated controls, then infectivity was gradually lost by minute by minute. At the very end, 10 min UV treatment completely inhibited infection of HeLa cells (Figure 5b).

#### 3.4.3. Acid and Alkaline Treatment

The effect of pH on viral infectivity in HeLa cultures was studied at different pH values between 1 and 14 at 25 °C and 37 °C. The results obtained at the two temperatures practically overlapped; therefore, the data obtained at 25 °C are shown here. Acidic pH 1 and 2 completely destroyed infectivity, followed by a gradually increasing residual capability for infection until the environment became pH neutral. Viruses remained stable between pH 7 and 10. To our surprise, pH 9 had an increasing effect on virus replication as compared to untreated controls, that is, more hexon antigen-positive cells were detected by immunochemistry. An alkaline pH around 11 and 12 resulted in a sudden drop (approximately 80%) of infectivity, while a very alkaline environment (pH 13 and 14) completely prevented infection of HeLa cells (Figure 5c).

#### 3.4.4. Effect of Disinfectants

Ten-fold dilutions of the stock solutions at 40 g/L NaClO and 650 mg/L of ethyl alcohol containing alkyl-dimethyl-ammonium-chloride were prepared; subsequently, aliquots of virus stock were introduced into these preparations. To avoid the toxicity of concentrated disinfectants, dilutions from 0.4 g/L NaClO and 6.5 mg/L ethyl-alcohol concentrations upwards were tested on HeLa cells. Residual virus infectivity gradually decreased towards the more concentrated solutions, but infection was partially retained at 0.4 g/L NaClO and 6.5 mg/L ethyl-alcohol concentrations. Finally, two-fold dilutions were prepared from the original stock solutions, and virus infectivity was tested as above. Complete loss of infectivity of the FeAdV isolate after 5 min incubation was seen at 5 g/L (equal to 0.067 mol/L) NaClO and at 65 mg/L (1.412 mol/L) ethanol solution, as no infected cells were found by immunochemistry.

#### 3.4.5. Antiviral Drugs

Preliminary tests were carried out using ten-fold dilutions of the stock solutions of each drug. Here, ribavirin at a final concentration of 0.5 mg/mL could not prevent virus replication in HeLa cells. A lower number of infected cells was seen after treatment with 1.0 mg/mL cidofovir and stavudine, although inhibition was not complete. It seemed practical to narrow the concentration range for antiviral drugs (ribavirin: between 5 and 0.5 mg/mL; cidofovir and stavudine: between 10 and 0.1 mg/mL). Two-fold dilutions were prepared for each drug, then HeLa cells were pretreated with these drug solutions for 1.5 h. Fifty-percent inhibitory concentrations (IC_50_s) were found with 0.625 mg/mL ribavirin or cidofovir and 0.3125 mg/mL stavudine in the pre-treatment regimen (Table 3).

Antiviral treatment of cells at 2 h post-infection resulted in the same effect. If antiviral treatment occurred at 24 h post-infection, higher amounts of drugs were required to reach the IC_50_ (e.g., 25.0 mg/mL ribavirin). The two other drugs proved to be more effective: cidofovir at 1.25 mg/mL and stavudine at 0.625 mg/mL exerted 50% inhibition of viral replication (Table 3).

### 3.5. FeAdV Infection Modifies Cytokine Release

Secretion of both regulatory cytokine proteins showed profoundly altered patterns through the course of viral replication as compared to the fluctuation in constitutive release from uninfected cells.

**Figure 6:**
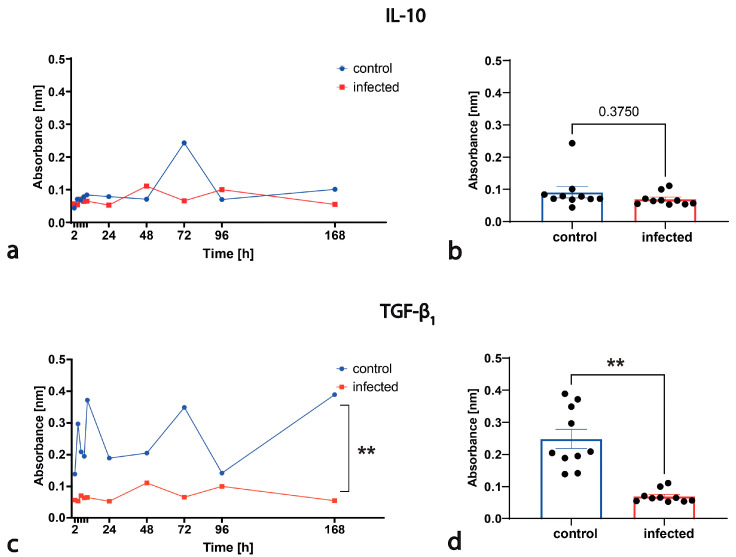
(**a**) The pattern of IL-10 secretion by mock-infected (blue) and FeAdV isolate-infected (red) HeLa cells at different time points. (**b**) Summary of IL-10 secretion after FeAdV isolate infection compared to mock-infected HeLa cells. All dots represent a different time point (n = 10). (**c**) The pattern of TGF-β_1_ secretion by mock-infected (blue) and FeAdV isolate-infected (red) HeLa cells at different time points. (**d**) Summary of TGF-β_1_ secretion after FeAdV infection compared to mock-infected HeLa cells. All dots represent a different time point (n = 10). Non-parametric t-test was used. All data are shown as means ± standard errors of means (SEMs). ** *p* < 0.001.

A minimal fluctuation in IL-10 production from control cells was seen during the culture period, except for a sudden peak at around 72 h. FeAdV isolate infection resulted in two small peaks at 48 and 96 h, and the monophasic constitutive peak disappeared (Figure 6a). The total amount of IL-10 protein secretion in mock-infected and infected cultures hardly differed (Figure 6b).

Constitutive TGF-β_1_ polypeptide secretion fluctuated widely during the cell cycle; practically four peaks were detected. Among the peaks, similar polypeptide levels were measured, with the lowest value at around 96 h. Viral infection resulted in marked suppression during the whole course of virus replication (Figure 6c). Total TGF-β_1_ secretion during the whole course of viral replication was significantly less than the total constitutive production (Figure 6d).

## 4. Discussion

### 4.1. Zoonosis

In the past several decades, numerous novel AdV genomes have been identified in a wide range of animals. Cross-species transmission of viral pathogens is becoming a public health concern. If a human host is involved, zoonosis or reverse zoonosis might occur. Animals in captivity or companion animals are vulnerable to novel opportunistic pathogens present in their anthropogenic environment [4,38]. AdVs are known to be extremely stable in the environment, suggesting that they may have multiple transmission routes [4]. Following recovery, HAdV shedding for several weeks also contributes to reverse zoonotic infections [8]. Recently, a number of adenoviruses have been described as reliable markers of fecal contamination in the environment [39]. HAdVs can be ingested and eliminated in the feces but not replicated in the guts of animals [40]. Detection of AdVs in several organs indicates a systemic infection [32]. Repeated positivity of fecal samples excluded the detection of accidental infection in our cat or laboratory contamination [26]. Successful isolation and sequencing showed the relatedness of the FeAdV isolate to the HAdV-C1 type [9,27]. This raises the speculation of a reverse zoonotic infection. The source of AdV infection in this cat and other cats screened for seroconversion [24,25] could have originated from pet owners or other humans, but it remains unknown. Other examples discussed below refer to interspecies transmission. HAdV-E4 was the first HAdV identified with a recombinant zoonotic origin, and HAdV-B76 was shown to be a recombinant that originated from human, chimpanzee and baboon AdVs, suggesting back-and-forth interspecies transmission events [8]. In stool specimens from fur seals, pampas foxes and crab-eating foxes, genes with a high nucleotide identity to the *pol* gene of HAdV-C were found [40,41]. Types of HAdV-C species establish persistent infection in humans; they are frequently shed symptomless into the environment [32], so there is a possibility to infect other humans and animals. A series of diagnostic tests overlapping with our attempts have been used to identify mastadenovirus DNA in a polar bear. Virus DNA and antigens were detected in host tissues and in some cell cultures (e.g., CRFK) but not in others (e.g., HeLa and HEK-293) without CPEs. Virus particles were not shown by electron microscopy. Such cases might suggest persistent infection. No established species-specific reagents could be used [4]. A new skunk AdV (SkAdV-1) was isolated and cultivated in liver cell cultures (HUH.7 and HEpG2), but not in CRFK and bat cells. Its penton base did not contain an RGD motif, similar to canine adenovirus type 2 (CAdV-2) [42] and human mastadenovirus YGD sequences [43], suggesting an RGD-independent cell entry. WGS showed its phylogenetic relation to bat AdV-2 and CAdV-1 [42]. The permissivity of cells, e.g., that of CRFK, might help set up in vitro models to study AdV pathogenicity. CRFK cells are also permissive for FIV, providing an in vitro model to study retrovirus–AdV interaction [34]. Cats are sensitive to several pathogenic human microbes [44], including subtypes of influenza A and SARS-CoV-2 [38]; these can also be studied in feline models.

In spite of technical shortcomings, such as persistent infection in vivo, limited cell-culture permissivity, lack of species-specific reagents, unidentified specific virus ligand molecules and partial genome sequences, accumulating data support the existence and phylogenetic relationships among newly discovered AdVs, including those in felids.

### 4.2. Sequencing

The large quantity of infectious virus particles of the FeAdV isolate obtained in permissive cell cultures provided an opportunity for biological and molecular characterizations. The complete genome sequence of FeAdV showed high nucleotide (99.997%) and amino acid (>99%) identity with the HAdV-C1 reference strain [12]. The first SNP alteration is an insertion of an A at the nucleotide position at 14,096, located between the peripentonal hexon-associated protein IIIA (12,327–14,084 nt) and the poly-A signal for gene L1 of HAdV-C1 (14,107–14,112 nt) [3]. The second SNP alteration in the genome of the FeAdV isolate was detected at nucleotide position 15,082 (corresponding to nt 15,081 in HAdV-C1), at nucleotide position 915 and amino acid position 305 in the penton protein-coding gene (L3, 14,166–15,890 nt) [3]. A T-to-C mutation did not result in an amino acid change (glycine). This amino acid is found in the RGD loop (298–373 aa) as compared to the published structure of HAdV-C2 [45]. The structure of the penton base is considered to be crucial in binding to integrins and clathrin-mediated endocytosis in mammalian cells [46].

It is of note that at the time of identification of the FeAdV isolate and sequencing of its hexon and fiber genes, WGS of HAdV-C1 was not available. Sequences of these genes were compared to the HAdV-C1 isolate 1038 obtained in the Netherlands and sequenced by us. The phylogenetic position of the FeAdV isolate was determined upon comparison with published partial gene sequences of other HAdV-C1 isolates. The consequences of hexon and fiber gene and amino acid alterations have been thoroughly discussed [9]. It can be mentioned here that the recent WGS and preliminary hexon sequence overlap, except in one nucleotide position (T to C in the variable region), with no amino acid alteration (Table 1). Sequencing the FeAdV isolate directly from the feces of the donor cat [26] showed two C-to-T, one T-to-C and one G-to-A alteration—all of them in the variable hexon region. The last variation resulted in a valine-to-methionine change. As compared to HAdV-C1, the early fiber sequence exhibited three nucleotide alterations without amino acid alterations (Table 2).

One of the limitations of our study is the detection of nucleotide differences in the partial- and whole-genome sequencing carried out on different FeAdV isolate stocks, in different laboratories and at different time intervals. For whole-genome sequencing, the FeAdV isolate from an early passage was stored in liquid nitrogen for several years and later revived and always propagated in HeLa cells. For the preliminary hexon and fiber sequencing, the virus was propagated in PD-5 porcine cells through several passages and only finally in HeLa cells again. It is possible that mutations occur and accumulate more frequently in PD-5 cells than in HeLa cells. Limited differences in the nucleotide and amino acid sequences of the HAdV-C1 prototype, isolate 1038 and the FeAdV isolate do not meet the requirements of a novel genotype of FeAdV; rather, the FeAdV isolate can be regarded as a variant of HAdV-C1.

### 4.3. Receptors

Partial sequencing and phylogenetic analysis of the hexon and fiber genes of the FeAdV isolate suggested that this isolate might be related to HAdV-C1. It was supposed that FeAdV binds to CAR and that its entry is mediated by integrin molecules. Antireceptor and coreceptor blocking reagents could be used to test this hypothesis. These antibodies are known to bind to human cells, and their avidity to bind to feline cells has not been tested, but feline-specific antibodies are not available. Simultaneous blocking of all three molecules suppressed infectivity in HeLa, CRFK and HEK-293 cells in the highest ratios. Blocking CAR and only one of the integrin molecules resulted in a less efficient reduction in permissivity, but the ratio showed minor cell-type differences. The FeAdV isolate has minimally stronger affinity to the α_v_β_3_ coreceptor on HeLa cells than to α_v_β_5_ molecules. On the contrary, FeAdV isolate particles minimally prefer α_v_β_5_ coreceptors on HEK-293 cells. The difference of the FeAdV isolate in coreceptor binding to CRFK cells is minimal; however, the feline-specificity of antibodies is unknown. HeLa cells are regarded as some of the most permissive cells for AdV infection. They express high levels of integrin α_v_β_5_ but little or no α_v_β_3_. Integrin α_v_β_5_ is widely expressed on several cells, whereas α_v_β_3_ is expressed in restricted cells. The RGD sequence has a low affinity for α_v_β_5_, although the RGD binding site containing adjacent single or multiple substitutions at the C terminus [47] might increase the affinity of RGD to α_v_β_5_ integrin. Integrin α_v_β_5_ binding facilitates membrane permeabilization of the endosome [48], accelerating AdV replication. HEK-293 cells express low levels of α_v_β_3_ and α_v_β_5_ integrins, while they express integrin α_v_β_1_, which also aids the internalization of AdV [49,50]. CRFK cells have been shown to bind and support AdV replication, especially C types of different animals, as discussed above. These cells express integrin α_6_, but the expression of other integrin types has not been tested yet [51]. The putative polar bear AdV antigens were shown in CRFK but not in HEK-293 cells [4], suggesting that integrin molecules can select the permissivity of cells for AdV infection. Accumulating evidence suggests that AdV penton bases can directly bind to integrin molecules. Leukocyte integrin α_M_β_5_ adheres to AdV, but α_v_ integrins are required for internalization. Integrin α_3_β_1_ can bind to HAdV-C5. Heparan sulphate gylcosaminoglycans (HS-GAGs) and lactoferrin can act as bridges between viral fibers and cell surfaces. In vivo, the infection of liver cells is CAR-independent; instead, it depends on AdV hexon binding to the blood coagulation factor X (fX). A series of cancer cells which express little to no CAR were pre-incubated with integrin-blocking antibodies subsequently infected with a recombinant HAdV-C5. Blocking integrin α_v_β_5_ dramatically prevented infection, while blocking β_1_ or α_v_β_3_ integrin on MCF-7 breast cancer cells moderately inhibited infection. These data also suggest that AdVs can use multiple entry routes [50]. Cancer is one of the most common targets of AdV-mediated gene therapy. CAR expression is often lost as cancers progress, and this loss is viewed as a major hurdle in the use of AdV-based vectors. Many AdV vector constructs bind directly and are internalized by integrin molecules, including PCa cells [52]. Our results also suggest that simultaneous blocking of receptor and coreceptor molecules could not completely prevent infection, supposing that particles of the FeAdV isolate can bind to and enter cells through alternative routes, as discussed in a preceding publication [9].

### 4.4. Physico-Chemical Effects

The highest permissivity for the FeAdV isolate in HeLa cells prompted us to study the effect of environmental conditions on its replication in this culture. Several new genotypes discovered recently have not been isolated; consequently, their biological characteristics have not been explored. Our studies on the physical integrity of the FeAdV isolate in different environmental conditions contribute to the understanding of its pathomechanism. Our results on the effectiveness of disinfection can be utilized in handling contaminated objects in veterinary surgery to prevent nosocomial infections as well as exposure of laboratory personnel through diagnostic procedures or producing adenovirus-based reagents and vectors [53]. In general, AdVs are resistant to ether and chloroform but lose their infectivity within 30 min at 56 °C and are inactivated by UV, 0.25% sodium dodecyl sulfate, and 0.5 μg/mL chlorine and formalin [5]. Different types exhibit many variations in resistance to harsh conditions.

#### 4.4.1. Heat

Applying heat treatment, namely, 56 °C, the FeAdV isolate was denatured in a very short time, within 3 min. Our result correlates with previous reports showing a rapid thermal denaturation, usually within 10 min, but heat resistance could be very divergent. HAdV-A12, -B14 and -A18 lose 99% of their infectivity in 8 min, while HAdV-C6 retains its stability at 56 °C for 10 to 15 min [36,37]. HAdV-B7 is inactivated at 50 °C within 26 min [54], HAdV-C5 after 10 min [53]; in another study, it was inactivated at 50 °C after 5 min [55]. HAdV types C1, C2 and B3, but not E4, lost infectivity at 50 °C after 10 min or at 56 °C after 2.5 min [56]. At 56 °C or a higher temperature, capsid polypeptides undergo denaturation; consequently, penton bases disintegrate and the fiber and attached hexon molecules separate [53,55,57]. The heat sensitivity of the FeAdV isolate is similar to that of human types 1, 2 and 3, but FeAdV seems to be more sensitive to heat than other human types. When handling specimens, the stability of AdV particles might be modulated by several factors (ionic strength, divalent cations, pH, time, the buffering capacity of solutions, age and the previous history of the specimens) [58]. The feces from which FeAdV was isolated were kept at different temperatures until the samples were processed. The cat had no fever; the feces were transported to and filtered in the virology laboratory at room temperature. HeLa cultures were infected at 37 °C. The FeAdV isolate did not disintegrate; consequently, both the PCR and isolation were successful.

#### 4.4.2. UV

Damage to the double-stranded DNA genome is the primary mechanism of inactivation by UV irradiation. Relevant studies focus on drinking-water safety. A 90% inactivation of HAdV-C2 was achieved by 40 mW/cm^2^, while 99.9% inactivation was achieved by 160 mW/cm^2^ UV irradiation. HAdV-C2 is more resistant to UV irradiation than the types F40 and F41 [59]. Approx. 80 mJ/cm^2^ low-pressure or 50 mJ/cm^2^ medium-level UV doses resulted in almost complete inactivation of HAdV-C2 [60]. Experiments on types C1, C2, C6 and F40 demonstrated 99.99% inactivation with irradiation doses of 137.9, 160.0, 153.8 and 217.1 mJ/cm^2^, respectively [61]. Experimental conditions that were more similar to those of our studies showed that 10 min irradiation of HAdV-D8 resulted in a complete loss of infectivity [36]. The resistance of the FeAdV isolate to UV irradiation is similar to that of other C types. As C types have usually been shown to cross host barriers, it seems that C species AdV types, including FeAdV, are more resistant to UV inactivation than other types tested. Due to climate change, AdV C types in animal carcasses and wastewaters can resist the more aggressive UV-B radiation of sunshine. Not in households, but where cats are kept in kennels or animal houses for experimental studies, more effective UV disinfection would be desirable. In nature, viruses are exposed to simultaneous causes of damage. The efficacy of UV disinfection is increased in the presence of chlorine; 50 mJ/cm^2^ or less leads to inactivation when both low-pressure (LP) and medium-pressure (MP) UV are used [62,63]. When 20 mJ/cm^2^ low-pressure and 10 mJ/cm^2^ high-pressure irradiations were combined with 0.17 mg/L free chlorine for HAdV-C2 disinfection, significant inactivation was achieved in a very short time (approx. 1.5 min) [62]. Another report stated that 40 mJ/cm^2^ or 73 mJ/cm^2^ UV doses are not enough to inactivate HAdV-F40 or -F41 without chlorine dioxide [64]. These reports suggest that inactivation of AdVs by combined methods could be more economical.

#### 4.4.3. pH

In general, AdVs have been found to be stable over a wide, even unfavorable pH range, e.g., between pH 3 and 12 [5,37], although several variations have been described. HAdV types A12, B14 and A18 are the most stable at around pH 6 and completely inactivated within 10 days at pH 4 and pH 8 [37]. HAdV-C5 is the most stable at pH 6, but at pH 5 its infectivity rapidly wanes [65]. The physical integrity of HAdV-C5 is improved between pH 5 and 8 [66]. Canine AdV (CAdV)-1 was found to lose infectivity at pH 9 more rapidly than at pH 7 in a fecal sludge [67], in sharp contrast to the stability of the FeAdV isolate at pH 9. Resistance to alkaline conditions is specific to felid AdV. Tomcats frequently struggle and bite each other; consequently, their alkaline saliva transmits FIV [21] and the FeAdV isolate directly into the bloodstream of fellow tomcats. All cats with suspected adenovirus infections, including ours, suffer from hepatic failure. AdVs can be excreted from the liver to the alkaline gut through bile, finally ending up in the near-neutral feces. Very early studies on HAdV-C1, C2, B3 and E4 reported highly overlapping results with ours: these types are the most stable between pH 6 and 9, while below pH 2 and above pH 11 they lose 99.99% of their infectivity [56]. Resistance to low pH has been shown to confer stability on gastric and biliary secretions; consequently, infective particles can be shed in the feces, promoting their environmental circulation [2]. Acidification can influence thermostability. HAdV-C5 particle morphology remained intact after one hour of pH 4 treatment at 4 °C and 36 °C. At 48 °C, virions appeared to be significantly disrupted at pH 7.8, 6.0, 5.5 and 4.0. At neutral pH, HAdV-F41 is more thermostable than HAdV-C5 [58]. The FeAdV isolate remains stable between pH 7 and pH 10 at 25 °C and 36 °C. Different noxae and their combinations can destroy cell-free AdVs, namely, FeAdV isolate particles, similarly to HIV-1. On the contrary, lymphoid cells in culture carrying chromosomally integrated HIV-1 survived acidification from pH 7.4 to pH 4.9 and regained virus production after neutralization [68]. Cells carrying AdV episomes latently might also survive environmental effects, and they can act as protective vehicles to transmit FeAdV infections to susceptible hosts.

After attachment to cell receptors and crossing the cell membrane, non-enveloped viruses hijacking the host endocytic mechanism are exposed to low pH (pH 6). This destabilizes AdV particles, disintegrates peripentonal hexon molecules and triggers AdV uncoating [58], followed by escape into the cytosol. IL-10 signaling raises the pH of endosomes, thereby diminishing the activity of acid-sensitive proteases that participate in antigen processing and AdV uncoating [69]. When the FeAdV isolate diminishes IL-10 production, the pH shifts towards acidity in the endosomes, which also enhances disintegration of the capsid.

#### 4.4.4. Chemicals

Adenoviruses are very resistant to several chemicals. Surface disinfectants containing 85–95% ethyl alcohol and 0.5 μg/mL NaOCl can inactivate adenoviruses within 10 min [5]. Chlorine efficiently and rapidly abolishes their infectivity. Recombinant HAdV-C5 was inactivated by using 0.2 mg/L free chlorine for 20 s and 0.5 mg/L free chlorine for 7 s [70]. Nascent chlorine rapidly inactivates HAdV-B3, depending on its concentration, pH and temperature, e.g., at pH 7, 25 °C and 0.08–0.12 ppm of ions destroyed 99% of viruses in less than 16 s [71]. Studies on HAdV-C2 showed that the minimum effect was achieved with 2.6 mg/L chlorine at pH 10 and 1 °C [72] or with 1.65 mg/L chlorine [73]. HAdV-C2, -F40 and -F41 in drinking water were destroyed by 0.2 mg/L free chlorine at pH 7 and 8 at 5 °C [74]. HAdV-C5 and -F41 lost infectivity at 22 mg/L chlorine at pH 8.5 after 5 min, while HAdV-F40 lost infectivity after 0.24 mg/L chlorine was used [75]. Combining chlorine disinfection with UV, HAdV-C2 was inactivated rapidly by doses as low as 10 mJ/cm^2^ MP UV and 0.17 mg/L free chorine at 5 °C within about 5 min [62], and HAdV-C5 was inactivated by 50 mJ/cm^2^ UV irradiation plus 0.15 mg/L chlorine [63]. The FeAdV isolate seems to be more resistant to disinfectants containing free chlorine than other HAdV types. Further experience regarding AdV resistance to disinfectants has been obtained through clinical practice. Disinfectant combinations containing 65% ethanol and 0.63% quaternary ammonium compounds, 79.6% ethanol plus 0.1% quaternary ammonium compounds, and 70% ethanol efficiently destroyed HAdV-C1 and HAdV-D8 within one minute [76]. HAdV-C5 is inactivated by solutions of ethanol between 70% and 90% in 30 s, while 2 m/m% ethanol in a gel can inactivate it in 2 min. HAdV-C2 seems to be more resistant, as a combination of 73.6 m/m% ethanol and 0.2 m/m% per acetic acid could destroy it in 30 s, but 55 m/m% or 85 m/m% ethanol solutions were required for complete disinfection. HAdV-B7 lost infectivity after exposure to 72.5–77 m/m% ethanol for 1 min, but the same parameters could not destroy HAdV-D8 [77]. We can conclude that our FeAdV isolate is very sensitive to detergents. These compounds completely destroy this virus at less than the usually applied concentrations and in a shorter time.

#### 4.4.5. Drugs

After seven decades of AdV research, there is no clinically approved treatment for AdV infections. Their sensitivity to antiviral drugs also varies widely. Broad-spectrum antivirals, such as ganciclovir, ribavirin and cidofovir, are commonly used, despite concerns about significant toxicities [8]. Ribavirin is known to inhibit replication of HAdV-C types only. In tissue cultures, 104 μM (0.02540 mg/mL) inhibited types C1 and C5 by 99%, while 111 μM (0.0271 mg/mL) inhibited HAdV-C2. Clinical isolates were inhibited at 99% by 229 μM (0.05592 mg/mL) and 155 μM (0.03785 mg/mL) ribavirin [78]. Similar results were presented by others on HAdV types C1, C2, C5 and C6 [79]. In contrast to the above findings, 50% inhibition of the FeAdV isolate was achieved by 1.25 mg/mL ribavirin pretreatment and 2 h post-infection treatment, but 5 mg/mL could inhibit viral replication at 24 h post-infection treatment. These results show that the feline AdV isolate is more resistant to ribavirin than several human types.

Cidofovir has been shown to be effective against a number of DNA viruses in cell cultures, animal models and clinical practice [80,81,82]. EC50 values were shown for cidofovir between 4.6 and 17 μg/mL [83]. Similar efficacy on HAdV types C1, C2, C5 and C6 was exhibited with 67 μM (18.7 μg/mL), 17 μM (4.7 μg/mL), 23 μM (6.4 μg/mL) and 27 μM (7.5 μg/mL), respectively. HAdV-C1 seems to be the most resistant [6]. A study found the 50% inhibitory concentrations for HAdV-C5, -D8 and -D17 to be 9.5, 4.7 and 5.4 μg/mL, respectively. HAdV-5 was the most resistant [82]. In another study, 50% inhibition was observed around the following concentrations: HAdV-B3: 0.5584 μg/mL, HAdV-B7: 0.3630 μg/mL, HAdV-D8: 0.2792 μg/mL, HAdV-A31: 0.3909 μg/mL and HAdV-C5: 0.1396 μg/mL, the latter being the most sensitive [84]. Compared to these varying results, both pre-treatment of cells and treatments post-infection equally required higher concentrations of cidofovir for 50% inhibition of the FeAdV isolate.

Among the antiviral drugs tested here, our FeAdV isolate was found to have the lowest resistance to stavudine. This result is unique with respect to former publications: 0.50 μg/mL for HAdV-B3, 0.091 μg/mL for HAdV-E4, 0.99 μg/mL for HAdV-D8, 5.7 μg/mL for HAdV-D19 and 5.3 μg/mL for HAdV-D37 were reported as EC50 values [85]. Stavudine at concentrations of 4.4 ± 0.5, 3.1 ± 0.4 and 0.69 ± 0.04 mM caused 50% inhibition of HAdV-C5 production after 2, 3 and 7 days of treatment in A549 cells [86]. As the FeAdV isolate is similar to HAdV-C1, this is the first report on the sensitivity of a HAdV-C1-related virus to stavudine.

In our study, the same concentration of drugs inhibited the replication of the FeAdV isolate if antiviral treatment occurred before or 2 h after infection, but treatment at 24 h post-infection required a four-fold higher concentration of ribavirin and a two-fold greater quantity of stavudine and cidofovir for 50% inhibition of FeAdV isolate replication. Our experimental data can be utilized if an AdV infection is diagnosed in veterinary or human clinical settings or unwanted virus replication can be stopped with vector-based applications.

### 4.5. Cytokines

AdVs exert a generalized effect on the host and elicit immune modulation. AdV-based expression vectors applied in 10^11^–10^13^ quantities in one shot can induce cytokine storm. Multiple efforts have been made to abolish the deleterious immune response. The major limitation of studying natural adenovirus infection in a small-animal model is that mice are not permissive to low-dose HAdV infection. After administering extremely high virus doses, the clinical scenario resembles that of immunocompromised hosts [1]. In vitro studies showed that induction of several cytokines, chemokines, along with protein kinases, transcription factors are elicited in a sequential manner after binding virus particles to CAR and other receptors (e.g., HS-GAGs) and RGD to α_v_ integrin coreceptors. In epithelial (e.g., A549 and HeLa) and immune cells, AdV internalization and delivery to the nucleus occurs in less than an hour, and during this time particle binding without viral gene expression elicits the secretion of pro-inflammatory mediators [1,13]. Activation or inhibition of other mediators might depend on AdV replication. The production of two important immunoregulatory cytokines, namely, IL-10 and TGF-β_1_, has been neglected.

IL-10 is expressed by many immune and epithelial cells. IL-10 plays a critical role in limiting the intensity and duration of the immune response by preventing overproduction of pro-inflammatory cytokines and chemokines [87]. Autocrine or paracrine production of IL-10 and TGF-β is one of the six known mechanisms of intratumoral immune evasion. Reports on IL-10 production in tumors and derived cell cultures are very controversial. IL-10 can stimulate cytotoxicity of tumor-resident CD8+ T cells but reduces CD8+ cell capacity to control pathogen burdens in chronic viral infections [88]. AdV E1 gene products can interact with several growth regulatory proteins participating in transcription control, cell cycle progression and apoptosis [2]. Fiber–CAR interaction was shown not to activate IL-10 transcription in A549 epithelial cells. AdV infection (also possibly by alternative virus entry) with a low virus burden on IL-10 secretion allows the production of other pro-inflammatory cytokines and increases CD8+ T cell cytotoxicity, while an extremely high AdV vector burden in the tumor microenvironment suppresses IL-10 production and does not limit the intensity and duration of the local immune response [13,89]. In a clinical trial, the combination of an oncolytic (Ad5-hTERT) AdV and IL-10 augmented CD8+ T cell-dependent antitumor efficacy [88]; in another clinical trial with an oncolytic AdV, CV787 increased blood levels of IL-1, IL-6, IL-10 and TNF-α [17]. IL-10 mRNA/protein is augmented in cervical cancer, but HeLa cells were reported not to express IL-10 [90]. The production of cytokines is usually tested at one undetermined time point during the viral replication cycle, as this was done in the above study. We wanted to improve the study of IL-10 secretion during the whole course of viral replication in the most permissive HeLa cell culture. We described a fluctuating constitutive IL-10 production pattern that is common with other mediator secretions. The replication of the FeAdV isolate profoundly suppressed the total IL-10 protein secretion. The same might happen in the body; several peaks during constitutive IL-10 secretion could be suppressed one by one if an AdV replicates continuously but not in a synchronized manner in mammalian cells.

We detected constitutive, continuous but fluctuating TGF-β_1_ secretion in HeLa cells. The FeAdV isolate significantly decreased its secretion; the high peaks were replaced by two small peaks at 48 and 96 h. The FeAdV isolate seems to suppress IL-10 and TGF-β_1_ secretion in a synchronized manner.

TGF-β_1_ is ubiquitously expressed in many types of cells. TGF-β_1_ possesses pleiotropic effects; it induces the activation, proliferation and differentiation of several other cell types. TGF-β_1_ acts in a biphasic manner because it is a growth inhibitor in normal epithelial cells but also promotes tumor growth, invasion and metastasis. All human tumors, particularly prostate cancers, overexpress TGF-β_1_. It inhibits the secretion and activity of many other cytokines, including IFN-γ and TNF-α, and downregulates IL-2 receptor expression, contributing to immune evasion. It is an endogenous factor that suppresses apoptosis in normal and pathological tissues [91,92]. HAdV-C5 12S mRNA production has been shown to repress the TGF-β_1_ promoter in HeLa cells [93]. Co-culture of cancer-associated fibroblasts (CAF) increased the expression of TGF-β_1_ and resulted in enhanced proliferation and colony formation, but decreased apoptosis of HeLa cells [94]. Several recent studies have focused on TGF-β_1_ gene silencing as an antitumor method. HeLa as well as Du145 human prostate cancer adenocarcinoma were infected with a defective AdV expressing human (h) and mouse (m) TGF-β_1_ and hTGF-β_1_. The greatest reductions in hTGF-β_1_ and mTGF-β_1_ mRNA levels expressed in HeLa cells and protein secretion were 89.7 and 76.5%, respectively [92]. Targeting TGF-β_1_ production by PCa and its metastases by conditionally replicating AdV vectors is a promising strategy to combat PCa [17]. Treatment of HeLa cells with different concentrations of TGF-β_1_ had no effect on cell proliferation at 24 h; however, after 48 and 72 h, TGF-β_1_ significantly inhibited the proliferation of HeLa cells in a time- and dose-dependent manner, and it inhibited apoptosis in a dose-dependent manner after 72 h of treatment [95]. The mechanism by which replication of the FeAdV isolate inhibits TGF-β_1_ secretion was not studied here. Activation of TGF-β molecules depends on a latency-associated peptide (LAP). Its RGD sequence binds to integrin α_v_β_6_ and weakly binds to α_v_β_5_. Inactivation of the β_6_ subunit gene downregulates inflammatory responses in mice [96]. Competitive binding of the penton base of the FeAdV isolate to integrins might interfere with TGF-β_1_ activation.

We were the first to demonstrate constitutive protein secretion expression of TGF-β_1_ by HeLa cells. Fetal bovine serum (FBS) used in the culture media of cells might contain small amounts of TGF-β proteins [92] that can increase threshold levels and be measured by ELISA, but the fluctuating level of TGF-β_1_ during cell culturing unambiguously proves its genuine production by HeLa cells. Our results, in agreement with the above molecular studies, suggest that natural AdV infection with inhibited TGF-β_1_ activity can restore antiviral immune response. Again, an extremely high virus burden after an AdV vector application itself can counteract tumor progression, which effect can be additive or synergistic with the delivered anticancer gene. This suggests that the construction of an oncolytic or vaccine AdV vector can exert immunostimulatory effects.

### 4.6. Limitations

One of the limitations of our study is that the pH of the feces of the donor cat was not measured. As discussed above, the pH of specimens might influence the success of virus isolation. The pH of feces of omnivores and carnivorous animals is usually higher than that of herbivores. Our cat was FIV-positive, and it is not known whether it was on a special diet. This experience suggests that in future the pH of feces ought to be measured if virus isolation is attempted.

Another remark is that suppression of IL-10 and TGF-β_1_ production upon FeAdV infection was not tested in PCa ongjos cells. It would be interesting to compare the pathophysiology of tumorous cells of different origins. HeLa cells do [97] but PCa ongjos cells possibly do not contain HPV E6 and E7 integrated genes, which can be confounding factors in cell metabolism and response to a heterologous infection. HPV has been shown to transactivate adenoviruses [98] that can contribute to the very high permissivity of HeLa cells for AdVs.

## 5. Conclusions

The classical diagnostic approach to identify AdV infections by isolation in cell culture and using antigen-based detection methods (e.g., enzyme immunoassays and latex agglutination) [39] has been replaced by nucleic acid-based procedures, mainly PCR and sequencing. Although the major advantage of these methods is the direct detection and classification of AdV in stool samples, the biological characteristics, pathogenicity and possible applications of a novel AdV for medical purposes (e.g., gene therapy) cannot be established. Cats seem to be an excellent animal model for studying AIDS, certain forms of leukemia and microbial interactions [21,44]. On the other hand, AdVs are established important pathogens in humans and the animal kingdom, except in Felidae. Following seroepidemiological and PCR-based surveys, we isolated an AdV from a cat. The isolate was first characterized by replication permissivity in cell cultures, antigen detection using specific human monoclonal antibodies due to lack of feline reagents, electron microscopy, and sequencing of its hexon and fiber genes in parallel with a Central European HAdV-C1 isolate [28]. Phylogenetic analysis showed that the first replication-competent FeAdV isolate is closely related to HAdV-C1 [9]. Here, we verified a nucleotide insertion in a non-coding motif and a nucleotide alteration without an amino acid change in the RGD loop of the penton protein-coding gene. These limited alterations suggest that the FeAdV isolate is a variant of HAdV-C1. Most probably, a reverse zoonotic infection from an unknown source occurred in the anthropogenic environment. It is not surprising that we isolated a variant of HAdV-C1 from a cat, in spite of the fact that cats and humans are phylogenetically very distant. It is of note that, among lentiviruses, feline immunodeficiency virus (FIV) is the only one which induces immunodeficiency in felids resembling HIV, including its molecular characteristics, furthermore, the pathogenesis and clinical course of human AIDS. A feline cell line, MBM, expresses several surface antigens that react with human monoclonal antibodies, as was tested before feline antibodies were available [99]. The peripheral blood leukocytes of the same SPF cats could also be immunophenotyped by using anti-human CD monoclonal antibodies (Supplementary Table S1) [100]. Later, human monoclonal antibodies were used for immunotyping in several feline clinical studies [101]. Other contemporary studies on feline AdV sero- and molecular epidemiology [24,25] suggest that cats can harbor AdVs of different origins. We have shown that, beside CAR and integrin α_v_β_3_ and α_v_β_5_ coreceptors, the FeAdV isolate can utilize alternative receptors and coreceptors for docking and entry into cells. Further exploration of the alternative receptor and coreceptor molecules could help identify specific targets on selected tumor cells in patients. We also studied permissivity in a recently established prostate cancer cell line. Studies on the secretion of two major regulatory cytokines through the complete replication cycle showed that the FeAdV isolate abrogates constitutive IL-10 and TGF-β_1_ secretion in HeLa cells. If an extremely large input of AdV vectors is introduced into a tumor mass, the lack of these important immunoregulatory peptides could indirectly contribute to the restoration of local antitumoral immunity. The only feline AdV isolate is more sensitive to heat, detergents and low pH, but is more resistant to alkaline and free chlorine conditions than HAdV types tested. Both clinicians and laboratory personnel ought to be informed about a new putative pathogen in cats. The usual handling and decontamination of feline carcasses and other biological samples seem to be sufficient to prevent AdV infection. The higher sensitivity to stavudine and increased resistance to ribavirin imply that stavudine or cidofovir could be applied in case of a suspected AdV infection in Felidae. Our studies provide an example of the search for and basic characterization of a novel AdV. Other feline AdV isolates would be required to elaborate specific diagnostic tools and explore pathogenic effects in pets and endangered large cats.

## Figures and Tables

**Table 1:** Comparison of sequence alterations at different stages of FeAdV isolate identification. brown: identical sequencing, green: nucleotide alterations, red: not identified nucleotide, blue: identical amino acid deduction, purple: an altered amino acid deduction.

Sequence length (bp)	36,001	35,990	2889	301	**Amino acid position**	**Location**		**AF534906 HAdV-C1 amino acid**	**PP259354** **FeAdV amino acid**	**AY512566 FeAdV hexon amino acid**	**AF172246 FeAdV partial hexon amino acid**
**Base**	**AF534906 HAdV-C1 base**	**PP259354** **FeAdV base**	**AY512566 FeAdV hexon base**	**AF172246 FeAdV partial hexon base**							
8888	deleted	A	nt	nt	2963	E2B		Lysine (+)	Lysine (+)	nt	nt
915	T	C	nt	nt	305	Penton proteine		Glycine (special)	Glycine (special)	nt	nt
81	G	G	G	N	26	Hexon gene	C1 5′-end	Glycine (special)	Glycine (special)	Glycine (special)	Glycine (special)
300	C	C	C	T	99		C1 5′-end	Tyrosine (polar)	Tyrosine (polar)	Tyrosine (polar)	Tyrosine (polar)
309	T	T	T	C	102		C1 recombinant region	Isoleucine (non-polar)	Isoleucine (non-polar)	Isoleucine (non-polar)	Isoleucine (non-polar)
315	C	C	C	T	104		C1 recombinant region	Glycine (special)	Glycine (special)	Glycine (special)	Glycine (special)
316	G	G	G	A	105		C1 recombinant region	Valine (hydrophobic)	Valine (hydrophobic)	Methionine (hydrophobic)	Valine (hydrophobic)
2877	T	T	C	nt	958		C1 recombinant region	Alanine (hydrophobic)	Alanine (hydrophobic)	Alanine (hydrophobic)	nt

nt: not tested.

**Table 2:** Comparison of fiber sequence alterations at different stages of FeAdV isolate identification. Brown: identical sequencing, green: nucleotide alterations, blue: identical amino acid deduction.

Sequence length (bp)	36,001	35,990	1749	**Amino acid position**	**Location**		**AF534906 HAdV 1 amino acid**	**FeAdV amino acid**	**AY518270 FeAdV fiber amino acid**
**Base**	**AF534906 HAdV 1 base**	**FeAdV base**	**AY518270 Fiber base**						
981	G	G	A	327	Fiber gene	17. pseudorepeat	Lysine (+)	Lysine (+)	Lysine (+)
1659	A	A	C	553		HI loop	Serine (uncharged)	Serine (uncharged)	Serine (uncharged)
1743	A	A	G	581		C-terminal	Glutamine (uncharged)	Glutamine (uncharged)	Glutamine (uncharged)

**Table 3:** The IC_50_ values of antiviral drugs affecting the infectivity of the FeAdV isolate during the pre- and 2 and 24 h post-treatments used during the study.

	Pre-Treatment	Treatment of Infected Cells	
		2 h Post-Infection	24 h Post-Infection
Ribavirin	0.625 mg/mL	0.625 mg/mL	25 mg/mL
Cidofovir	0.625 mg/mL	0.625 mg/mL	1.25 mg/mL
Stavudine	0.3125 mg/mL	0.3125 mg/mL	0.625 mg/mL

## Data Availability

The feline adenovirus genome sequence is available in GenBank under the accession number PP259354.

## Supplementary Materials

The following supporting information can be downloaded at: https://www.mdpi.com/article/10.3390/ani14233502/s1, Table S1: Immunophenotyping characterization of feline peripheral blood leukocytes by human monoclonal antibodies.
