# Supplementary material for: Feline Adenovirus Isolate Shows Silent Nucleotide Alterations, Alternative Receptor/Coreceptor Binding, High Resistance to Disinfectants and Antiviral Drugs, as Well as Immunomodulation
Source: Animals (Basel). 2024 Dec 4;14(23):3502. doi: 10.3390/ani14233502 (PMC11640234; doi:10.3390/ani14233502)
Supplement: Supplementary file 1 [file animals-14-03502-s001.zip › animals-3230687-supplementary.pdf]

Supplementary Table S1. Immunophenotyping charaterization of feline peripheral blood leukocytes by human monoclonal antibodies (in percentage of cells)

**Specificity of monoclonal antibodies**	**Peripheral blood leukocytes (%)**
CD2, CD3, CD8, CD22, CD25	1
CD1a, CD4, CD19, CD23, CD36	2
CD11b, CD15, CD38, CD45	3
CD71, HLA-DR	4
CD7	5
CD34	7
CD20	8
CD24	9
CD13	10
CD21	11
CD42b	12
MHC Class I	14
CD33	18
CD41a	19
CD56	30
CD57	32
CD14	39

Origin of SPF cats, drawing and separation of peripheral blood cells are described previously. Monoclonal antibodies were purchased from Becton Dickinson, San José, CA, USA (Matteucci et al. 1995). Flow cytometry analysis was carried out by Dr. A. Pinto and Dr. G. Marotta at „Centro Regionale di Riferimento Oncologico, Unita Operativa Leucemie", Aviano, PN, Italy in 1993.
